# Fitness effects of plasmids shape the structure of bacteria–plasmid interaction networks

**DOI:** 10.1073/pnas.2118361119

**Published:** 2022-05-25

**Authors:** Arthur Newbury, Beth Dawson, Uli Klümper, Elze Hesse, Meaghan Castledine, Colin Fontaine, Angus Buckling, Dirk Sanders

**Affiliations:** ^a^Environment and Sustainability Institute, University of Exeter, Cornwall TR10 9FE, United Kingdom;; ^b^Centre for Ecology and Conservation, Biosciences, University of Exeter, Cornwall TR10 9FE, United Kingdom;; ^c^Institute for Hydrobiology, Technische Universität Dresden, 01062 Dresden, Germany;; ^d^Centre d’écologie et des Sciences de la Conservation, CESCO, UMR7204, Muséum National d’Histoire Naturelle, CNRS, Sorbonne Université, 75005 Paris, France

**Keywords:** networks, antibiotics, plasmids, microbiology, ecosystems

## Abstract

Antimicrobial resistance (AMR) genes are often carried on broad host range plasmids, and the spread of AMR within microbial communities will therefore depend on the structure of bacteria–plasmid networks. Empirical and theoretical studies of ecological interaction networks suggest that network structure differs between communities that are predominantly mutualistic versus antagonistic, with the former showing more generalized interactions (i.e., species interact with many others to a similar extent). This suggests that mutualistic bacteria–plasmid networks—where antibiotics are present and plasmids carry AMR genes—will be more generalized than antagonistic interactions, where plasmids do not confer benefits to their hosts. We first develop a simple theory to explain this link: fitness benefits of harboring a mutualistic symbiont promote the spread of the symbiont to other species. We find support for this theory using an experimental bacteria–symbiont (plasmid) community, where the same plasmid can be mutualistic or antagonistic depending on the presence of antibiotics. This short-term and parsimonious mechanism complements a longer-term mechanism (coevolution and stability) explaining the link between mutualistic and antagonistic interactions and network structure.

## Significance

Antimicrobial resistance (AMR) poses a great challenge for modern medicine. Plasmids are important vectors of antibiotic resistance genes. Plasmids can have context-dependent effects on their hosts, generally slowing their growth rate but also providing protection from specific antibiotics and heavy metals. Thus, models that predict population densities based on interactions between species are useful for explaining plasmid dynamics. Here, we predict with a simple ecological model the properties of a host (e.g., bacteria) and symbiont (e.g., plasmid) interaction network. Using experimental microbial communities and a conjugative plasmid, we confirm our predictions that beneficial symbionts spread more widely through a microbial community and provide key experimental results for network ecologists seeking to uncover the determinants of ecological network structure.

The widespread occurrence of antimicrobial resistance (AMR) is a looming crisis for medicine (1). Mobile genetic elements (MGEs), such as plasmids, are important vectors of antibiotic resistance genes in the natural environment and in hospitals (2–4). To predict and control the spread of AMR-carrying plasmids, we need to understand the ecology of host bacteria and their plasmid symbionts (5, 6). Plasmids carrying AMR genes are often very abundant in microbial communities and shared by many hosts (7, 8), suggesting that plasmids that can confer net fitness advantages to their hosts are likely to have a higher ecological generality (the property of interacting with many species) in microbial communities than plasmids that impose net fitness costs (9). This pattern fits with predictions and observations from ecological network theory: mutualistic (both parties benefit from each other) interactions lead to better connected interaction networks with a larger proportion of generalist species than networks where interactions are antagonistic (10–14). Current understanding of the mechanisms shaping such differences in network structure has focused on two nonmutually exclusive hypotheses. First, coevolution can lead to the observed patterns under the assumption that trait matching (e.g., a virus’ receptor protein must match a host receptor) determines the strength of antagonistic interactions, while exploitation barriers (e.g., a pollinator’s mouthparts must be at least as long as a given flower’s corolla tube) determine mutualistic interactions (15–17). Second, greater generality can foster stability (notably, greater robustness against extinctions and population fluctuations) in mutualistic networks but potentially destabilize antagonistic networks (14, 18–20). Note that these mechanisms linking interaction type and network properties have yet to be experimentally demonstrated.

Here, we demonstrate with mathematical modeling and experimental microbial communities that the degree to which a plasmid spreads through a host community (both the number of host species in which it is found and the evenness of its prevalence between host species) can be simply explained as a direct result of the affect the plasmid has on its hosts’ population growth rate. Beneficial plasmids, carrying AMR genes, have a higher ecological generality, leading to a more connected network structure. The model used makes no assumptions specific to bacteria or plasmid biology, and hence our predictions should be broadly applicable to any host–symbiont system with both vertical and horizontal transmission.

## Results

### Theory.

First, we modeled the change in prevalence of a symbiont within a host population. Symbionts transmit horizontally (between free-living hosts), vertically (parent to offspring), or, in many cases, via both modes (21, 22). We assume the latter here. The spread of a symbiont through a population of potential hosts owing to vertical transmission depends on the infected hosts’ reproduction and death rates relative to the rest of the population. Thus, if we combine reproduction and death into a growth term ωA for infected hosts and ωB for uninfected hosts, we can model the change in frequency of infected hosts from one generation to the next using a discrete time haploid selection model (23)

$$\Delta p\;=p\left(1-p\right)\frac{\omega A-wB}{p\omega A\;+\;\left(1-p\right)\omega B},$$

where *p* is the frequency of infected hosts. Here, if wB > ωA, the symbiont will eventually be lost from the population as the right-hand side of the equation becomes negative. However, to account for horizontal transmission, we include the transmission coefficient β from susceptible-infected (SI) models to account for both the encounter rate and the frequency of transmission given an encounter between an infected and uninfected host, resulting in a change in infection frequency of βp(1−p) in a single generation (24), giving

[1] $$\Delta p\;=p(1-p)\left(\frac{\omega A-wB}{p\omega A\;+\;(1-p)\omega B}+\;\beta \right).$$

The same phenomena can be modeled in continuous time. Here, the horizontal transmission term remains the same, as this takes the same form in continuous time SI models (23). The continuous time haploid selection model takes the form dp/dt =p(1−p)(rA−rB) (23). Here, *rA* and *rB* are the growth rates of infected and uninfected hosts, respectively. Thus, the change in the frequency of infected hosts over time is given by the ordinary differential equation (ODE)

[2] $$\frac{dp}{dt}=p(1-p)(rA–rB+\beta ).$$

**Fig. 1.**
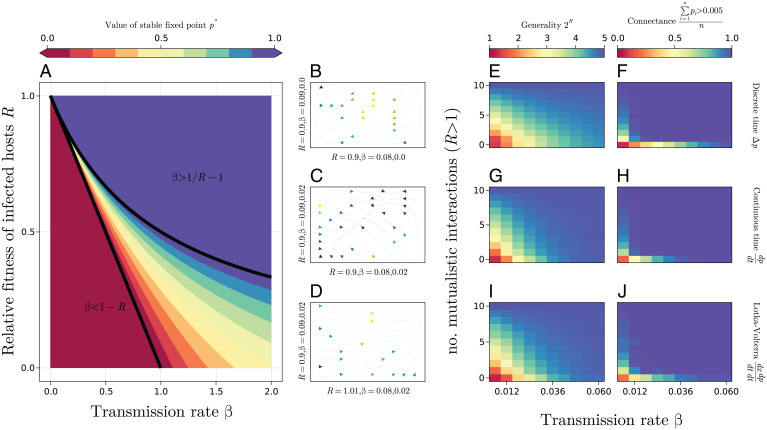
Numerical simulations show the power of symbiont-mediated host fitness effects to alter symbiont prevalence and host–symbiont networks. (*A*) The value of the stable fixed point in a single host species discrete time model. Equilibrium symbiont frequency is a function of transmission rate β and relative fitness of hosts R = ωA/ωB, where ωA and ωB  are the fitness of the host with and without the symbiont, respectively. Internal equilibria always and only exist when 1−R<β<1/R−1 (drawn in black) and are unique and globally stable when they do exist. Otherwise, there is a globally stable fixed point at 1 or 0 when β>1/R−1 or β<1−R, respectively. When *R* > 1 (i.e., mutualism) and when *R* = 1 and β > 0, the symbiont will always go to fixation. (*B*–*D*) Example trajectories of infection frequency in a two-host discrete time model. Each axis represents the infection frequency of a single host and is labeled with that host species *R*, followed by their intraspecific and interspecific transmission rates in that order. Brighter, yellow hues denote a faster rate of change. In *B*, with some intraspecific and no interspecific horizontal transmission, both host species lose the parasitic symbiont. The addition of a small amount of interspecific transmission leads to an internal equilibrium in *C*, with both host species having intermediate infection frequencies. In *D*, one host species derives a slight benefit from the symbiont, which then goes to fixation in both host species. (*E*–*J*) Results from numerical simulations, with communities of 10 host species and a single symbiont, in discrete time (*E* and *F*), continuous time (*G* and *H*), and continuous time with the addition of competitive interactions between host species (showing those instances where all host species survived; see *SI Appendix*, Fig. S6 for results with extinctions) (*I* and *J*). In each case, there is a large increase in connectance (proportion of species with prevalence > 0.005) when at least one host–symbiont interaction is mutualistic (R>1), but symbiont generality increases more gradually with the number of mutualistic interactions.

In Eq. **2**, the equilibria at *P* = 1 or 0 are clearly stable, unstable, or neutral depending on the sign of the second term (rA–rB +β). Additionally, all values of *p* are neutral equilibria when the symbiont is parasitic (*rA* < *rB*) and horizontal transmission rate is equal to the absolute difference between *rA* and *rB*. Conversely, in the discrete time model (Eq. **1**), unique, stable, internal equilibria occur at P= 1–1/R– 1/β, if and only if 1−R<β<1/R−1, where R=ωA/ωB is the fitness of infected relative to uninfected hosts (Fig. 1*A*; *Materials and Methods*). In either case (discrete or continuous time), the symbiont will always go to fixation when it is mutualistic (i.e., ωA >ωB  or rA> rB, respectively).

Expanding the model to account for multiple hosts, while still assuming the total population size of each species remains constant, gives

[3] $$\Delta p_{i}=p_{i}\left(1-p_{i}\right)\left(\frac{\omega A_{i}-wB_{i}}{p_{i}\omega A_{i}\;+\;\left(1-p_{i}\right)\omega B_{i}}\right)+\;\left(1-p_{i}\right)\underset{\beta_{ij}}{\sum }\beta_{ij}p_{j}$$

and

[4] $$\frac{dp_{i}}{dt}=p_{i}(1-p_{i})(rA_{i}\;–\;rB_{i})\;+\;\left(1-p_{i}\right)\underset{\beta_{ij}}{\sum }\beta_{ij}p_{j}$$

for discrete and continuous time, respectively, where $\beta_{ij}$ is the horizontal transmission rate from species *j* to species *i*. With multiple host species, small changes in fitness effects of the symbiont or transmission rates in one species can have a large impact on the infection frequency of other host species (Fig. 1 *B*–*D*). Expanding the scope of the multihost continuous time model to allow for fitness differences between species and interspecific interactions to affect host population densities, we assume that the strength of selection within a population is independent of population size, but horizontal transmission will depend on the density of both the donor and recipient population. The change in abundance of infected hosts in the *i*th species due to horizontal transmission is then $x_{i}\left(1-p_{i}\right)\underset{\beta_{ij}}{\sum }\beta_{ij}p_{j}x_{j}$, where $x_{i}$ is the population size of the *i*th species. Dividing through by $x_{i}$ to get the proportionate change, we model the change in population densities and the change in the frequency of infected hosts within populations as

[5.1] $$\frac{dx_{i}}{dt}=r_{i}x_{i}\left(1-\;\frac{\sum_{j}^{}\alpha_{ij}x_{j}}{K_{i}}\right)$$

[5.2] $$\frac{dp_{i}}{dt}=p_{i}(1-p_{i})(rA_{i}\;–\;rB_{i})\;+\;\left(1-p_{i}\right)\underset{\beta_{ij}}{\sum }\beta_{ij}p_{j}x_{j},$$

where *x_i_* is the density of the *i*th host species, *r_i_*_=_ *rA_i_*(*p_i_*) + *rB_i_*(1−*p_i_*), *K_i_* is the carrying capacity of species *i*, and *α_ij_* is the effect species *j* has on the population of species *i*. Thus, the first equation is a Lotka–Volterra competition equation (25), where growth rates depend on infection frequencies, and the fitness effects of the symbiont and population growth is reduced (potentially becoming negative) with the increase in size of all competing populations. Additionally, horizontal transmission to, from, and within species with small population sizes will be reduced. For each of the multihost models (Eqs. **3** to **5**), we simulated the dynamics of a 10-host–species community 100,000 times for 1,000 generations, varying all applicable parameters. Both connectance (the number of species the symbiont infects) and symbiont generality (connectance weighted by the evenness of infection, measured as Shannon’s diversity index of infected hosts) in the network increase with horizontal transmission rate and the number of host species that derive a benefit from the symbiont (i.e., the number of mutualistic interactions; Fig. 1 *E*–*J*). Even a single mutualistic interaction has a large impact on the number of links and generality. This is because the symbiont goes to fixation in its mutualistic host’s population, and this host species then acts as a hub, continually transmitting (parasitic) symbionts horizontally to the rest of the community. Despite differences in the modeling approaches (e.g., the differences in internal equilibria between continuous and discrete single-host models (Eqs. **1** and **2**) and the fact that with variable population densities (Eqs. **5.1** and **5.2**), a parasite (*R* < 1) can slow its own transmission by reducing its hosts population size), the results of the multihost simulations are all very similar. Thus, we show how a change between positive and negative fitness effects is sufficient for the establishment of different network characteristics.

### Experiments.

We experimentally test our predictions using a five-isolate (*Achromobacter* sp., *Stenotrophomonas* sp., *Ochrobactrum* sp., *Pseudomonas* sp., and *Variovorax* sp.) community of soil bacteria (26) and a conjugative plasmid (pKJK5::gfp) as the symbiont, modifying the abiotic context in order to alter the interaction sign between the plasmid and its bacterial hosts. The five bacterial species can coexist for longer periods than our experimental duration, each species can invade each other from rare, and most pairwise interactions are competitive, with the exception that *Variovorax* sp. which benefits from both *Ochrobactrum* sp. and *Pseudomonas* sp. (26). Conjugative plasmids are MGEs that rely on reproduction of and conjugation between bacteria for propagation (22). Due to metabolic costs they confer upon their bacterial hosts (27), plasmids are typically parasitic (28). However, like many plasmids, pKJK5::gfp (29) confers antibiotic (tetracycline) resistance. Consequently, the bacterial species have a mutualistic relationship with the plasmid in the presence of tetracycline, with the plasmid conferring resistance, and the bacteria facilitating plasmid reproduction (*SI Appendix*, Fig. S1). We cultured the experimental bacterial communities and plasmid pKJK5::gfp for 5 wk in the presence and absence of tetracycline. pKJK5::gfp was introduced by a single donor strain (*Ochrobactrum* sp., *Pseudomonas* sp., or *Variovorax* sp.). To control for the effect of plasmid prevalence in the initial hosts on subsequent interspecific conjugation rates, plasmid prevalence in the donor strain was initially 100%.

**Fig. 2.**
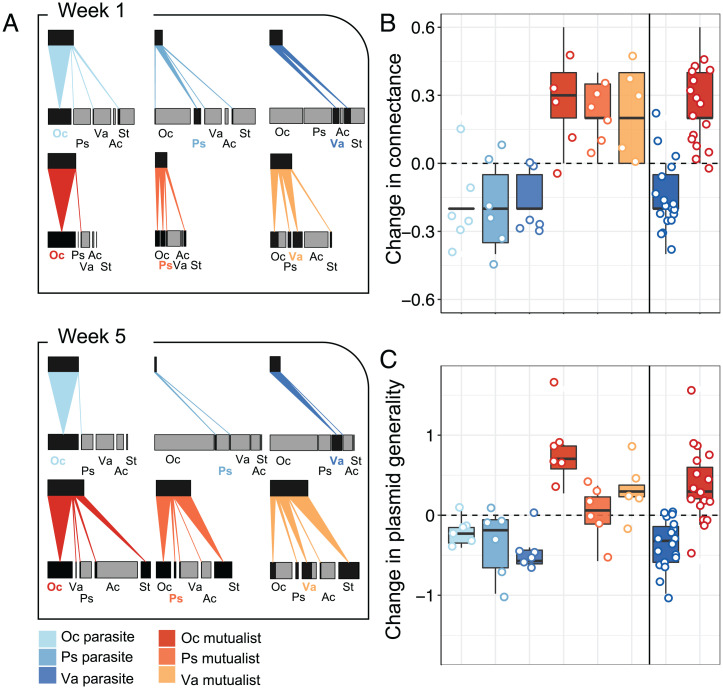
Experimental host–plasmid networks under mutualistic and parasitic interactions. (*A*) Bacteria–plasmid interaction networks in weeks 1 and 5 for the six treatments where *Ochrobactrum* sp. (Oc), *Pseudomonas* sp. (Ps), and *Variovorax* sp. (Va) serve as initial plasmid donor either with the plasmid as parasite (without tetracycline) or mutualist (with tetracycline). For each host species (lower bar labeled with the first two letters of their genus name), the proportion of plasmid-carrying cells is presented in black, and plasmid-free cells are in gray. Presented is the mean for abundance and interaction frequency per treatment (*n* = 6). The size of the bars indicates relative abundance within each week compared to the community with the highest abundance; Ac, *Achromobacter* sp.; St, *Stenotrophomonas* sp. (*B* and *C*) The change in network metrics for communities from week 1 to week 5 are presented with median, interquartile ranges, and single values for the different single treatments and overall parasitic and mutualistic treatments with connectance (*B*; realized number of links divided by the number of possible links) and plasmid generality (*C*; Table 1; *SI Appendix*, Fig. S3).

After 1 wk, there was little difference in the network properties between mutualistic and antagonistic networks, and the plasmid spread from the donor species to a small fraction of one to three other bacterial species in the community (Fig. 2*A*). After 5 wk, the plasmid was present in four to five species in the mutualistic networks, while it was only present in two species in the antagonistic networks (Fig. 2*A*). Consequently, both network connectance and plasmid generality significantly increased between weeks 1 and 5 in the mutualistic networks, while both decreased in the antagonistic networks (Fig. 2*B* and Table 1). Donor species-specific effects on network metrics in week 1 disappeared largely by week 5 (Table 1), resulting in consistent patterns within each treatment (Fig. 2 *B* and *C*). Our experiments match our theoretical predictions in that mutualistic symbionts reach higher prevalence, and this leads to more connected and generalized networks.

**Table 1.** Response of network metrics to experimental treatments

			Connectance		Plasmid generality	
	numDF	denDF	*F*	*P* value	*F*	*P* value
Intercept	1	30	**564.9051**	**<0.0001**	**1627.4368**	**<0.0001**
Tet	1	30	**47.4675**	**<0.0001**	**42.6037**	**<0.0001**
Donor	2	30	2.5486	0.095	**5.4224**	**0.0098**
Week	1	28	1.1134	0.3004	0.0002	0.9887
Tet × donor	2	30	**5.1161**	**0.0123**	1.307	0.2856
Tet × week	1	28	**42.7726**	**<0.0001**	**40.8071**	**<0.0001**
Donor × week	2	28	0.5232	0.5983	**6.5773**	**0.0046**
Tet × donor × week	2	28	0.1938	0.8249	**3.5081**	**0.0437**

Results from linear mixed effects models testing for treatment × time interaction on the network metrics connectance and plasmid generality. Tet, tetracycline. Test statistic and *P* value in bold where *P* < 0.05. numDF, degrees of freedom in the numerator; denDF, degrees of freedom in the denominator.

**Fig. 3.**
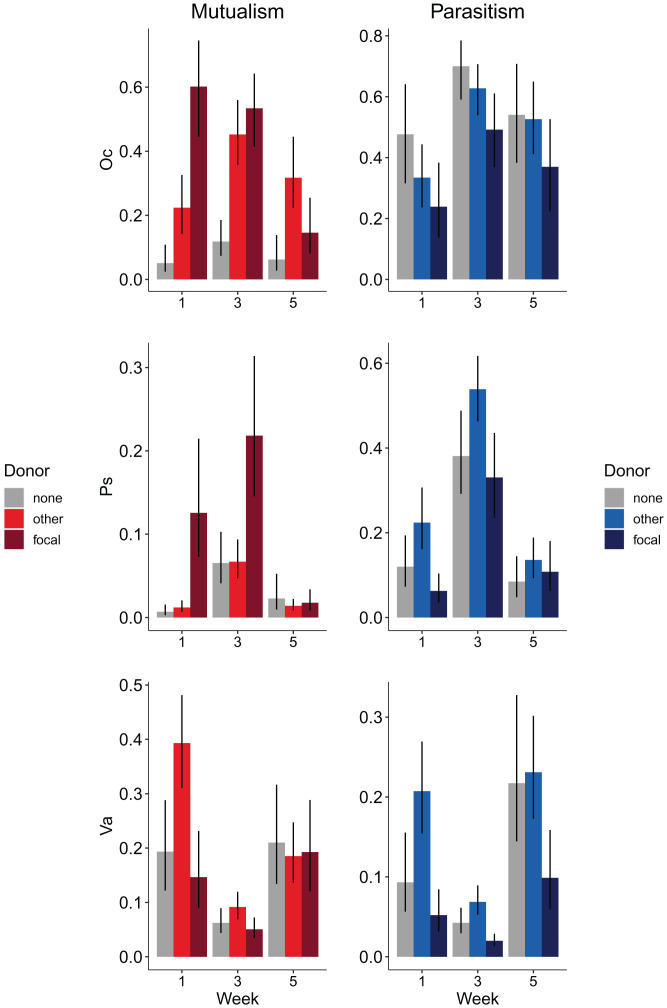
Fitness effect on the plasmid donor species. Relative abundances of the three plasmid donor species, *Ochrobactrum* sp., *Pseudomonas* sp., and *Variovorax* sp. (identified by abbreviated genus name) in treatments without plasmid (gray), when another species is the donor (light color), and when the focal species is the donor (dark color), in the presence (red) and absence (blue) of tetracycline. Bars show the mean ± 95% credible intervals extracted from the posterior distribution of glmer models (*Materials and Methods*). For statistical tests, see Table 1, and relative abundance of the whole community is in *SI Appendix*, Fig. S4.

The experimental results are also consistent with the predicted mechanism driving this pattern: the frequency of a host species infected with a symbiont within a community is determined by the relative impact the symbiont has on host fitness. When measured in monoculture, all isolates exhibited reduced growth rates when grown with pKJK5 in the absence of tetracycline (*SI Appendix*, Fig. S1). All three hosts reached lower frequencies in the community when they were the initial plasmid donors than when they were not (Fig. 3). In the presence of tetracycline, all but *Variovorax* sp., which showed the greatest constitutive resistance to tetracycline, benefitted from the plasmid (*SI Appendix*, Fig. S1). In the community context, both *Ochrobactrum* sp. and *Pseudomonas* sp. (but not *Variovorax* sp.) reach higher densities in week 1 when they were the plasmid donor than when they were not.

While our results fit our predictions, we conducted additional experiments to investigate other possible explanations for the observed patterns. In particular, it is possible that tetracycline had a direct impact on plasmid conjugation rates (for example, by altering patterns of gene expression [30]) or an indirect effect by altering total cell density and hence contact rate. To determine whether the concentration of tetracycline used in our experiment altered conjugation rates, we performed filter mating assays (*Materials and Methods*) with and without tetracycline. We detected a lower frequency of conjugation events in the presence of tetracycline (*SI Appendix*, Fig. S2), suggesting that direct effects of tetracycline on conjugation rates, if anything, caused a reduction in symbiont generality and connectance in mutualistic bacteria–plasmid networks. Additionally, we found no evidence of a positive or negative impact of cell density on either connectance or generality (*SI Appendix*, Fig. S5).

Our results can be readily explained by ecological dynamics. However, it is likely that some (co)evolutionary changes occurred in bacteria and plasmids, given that bacteria underwent in the region of 50 generations. We investigated the possibility that this may have contributed to the observed patterns. In particular, we considered whether increased conjugation rates may have evolved in mutualistic treatments. To this end, we investigated evolutionary changes in the community replicates that showed the greatest increase and decrease in connectance over time (a *Pseudomonas* sp. donor in an antagonistic treatment and an *Ochrobactrum* sp. donor in a mutualistic treatment, respectively). We measured the spread of the plasmid to the rest of community from the donor isolated at week 5 and ancestral donors over 1 wk in the absence of tetracycline. We found nonsignificant increases in connectance and generality in evolved (versus ancestral) *Pseudomonas* spp. and nonsignificant decreases for *Ochrobactrum* spp. These patterns are the opposite of those expected if (co)evolution contributed to the ecological patterns. That said, there was clear evidence of evolution. While all *Ochrobactrum* spp. were fully infected whether evolved or ancestral, the evolved *Pseudomonas* sp. had lower plasmid prevalence than the ancestor (5% versus 39%; quasibinomial general linear model [GLM; *χ*^2^_1_ = 267.21, *P* < 0.001]). The evolution of rapid loss of the plasmid may explain why *Pseudomonas* sp. was the only donor species that tended to reach higher relative density in the presence of the parasitic plasmid by week 5 (Fig. 3).

## Discussion

Given the propensity for plasmids to encode AMR genes, our results suggest that exposure of microbial communities—including human microbiomes—to antibiotics may increase plasmid–host network connectance and generality. This may not only alter the stability and composition of microbial communities but also result in network structures that facilitate the spread of other plasmid-encoded genes, including AMR genes and virulence factors, not under positive selection. Previous work using emulsion, paired isolation, and concatenation PCR (epicPCR) (31) to detect the presence/absence of bacteria–plasmid associations (at the strain level) reveals that the number of bacterial species that take up a resistance-conferring plasmid can be positively impacted by low concentrations of antibiotics (5, 9). We extend this by using a fluorescent marker to identify plasmid hosts at the individual colony-forming unit level and quantify the extent of plasmid spread throughout each species, providing a simple, parsimonious model and explicitly testing for effects not included in our model (antibiotics increasing conjugation rate and/or reducing total cell density) to show that differences in growth rate due to plasmid carriage lead to more generalist and better connected bacteria–plasmid networks (*SI Appendix*, Figs. S1 and S5). While we certainly expect total cell density to impact conjugation frequency and hence plasmid spread in general, the resistance conferred by the plasmid lead to similar cell densities across treatments. This is likely why we did not detect an effect of density on either connectance of generality.

Our results are relevant to host–symbiont interactions in general, especially under temporally or spatially varying environmental conditions that move a symbiont along the mutualist–parasite continuum (32). For example, warming ocean temperatures lead to a shift from mutualism to parasitism in coral symbiont systems (32), and in many insect–symbiont systems, regional variation in the presence of natural enemies (to which symbionts confer protection) (33) and abiotic factors such as temperature (34) lead to a geographic mosaic of interaction types within the same species pair. The simple mechanisms described here also likely apply to free-living species in mutualistic and antagonistic networks. An initially specialized mutualist will increase the population size of the species they interact with. This will in turn increase the population size of the mutualist they are interacting with, increasing exposure to other host species.

Here, we add an ecological mechanism leading to the contrasted network structure observed in mutualistic and antagonistic communities. We demonstrate that the fitness differences induced by mutualistic and antagonistic interactions during the early stages of network formation are enough to shape networks in contrasted ways. These short-term dynamics create the context for longer-term ecological and evolutionary dynamics (i.e., coevolution and stability) identified as mechanisms linking interaction type and network properties.

## Materials and Methods

All supporting data are available at https://zenodo.org/record/5342448, and associated analysis code is available at https://github.com/EvoArt/PlasmidNetworks.

### Model.

#### Stability of a single host species discrete time model.

In Eq. **1** the system is at equilibrium when selection balances transmission, that is, when

$$p=\;\frac{\omega B}{\omega B-\omega A}-\frac{1}{\beta }.$$

Feasible internal equilibria (i.e., where 0 < *P* < 1) exist only when infected hosts have lower fitness than uninfected hosts (i.e., when the symbiont is parasitic) and

$$0<\frac{\omega B}{\omega B-\omega A}-\frac{1}{\beta }<1.$$

Making use of the fact that all variables in the inequalities must be positive (we assume hosts are always capable of reproduction at some rate, and note that when *β* = 0, Eq. **1** is simply a haploid selection model), we can rearrange them to get

(ωB−ωA)/ωB<β<(ωB−ωA)/ωA.

Substituting *R* for *ωA*/*ωB* gives

1−R<β<1/R−1.

We use linear stability analysis to show that whenever internal equilibria exist, they are stable because both exterior equilibria will be unstable. First, adding *p* to Eq. **1**, taking the first derivative with respect to *p*, and substituting *P* = 0 gives *R* + *β*, which is > 1 when 1−R<β. Substituting *P* = 1 gives 1/*R* – *β*, which is >1 whenβ<1/R−1. Thus, wherever internal equilibria exist, both exterior equilibria are unstable, and the internal equilibrium is globally stable.

#### Numerically solving multiple host species models.

To predict the effects of interaction sign and transmission rate on symbiont generality, we implemented 100,000 numerical simulations of each of the multispecies equations (Eqs. **3** to **5**) in Julia (35) using DifferentialEquations.jl, with the continuous time ODEs (4, 5) solved by the fifth-order explicit Runge–Kutta method (36). In each simulation, 10 host species were assigned *ωA* and *ωB* from a random uniform distribution between 0 and 1. β values were drawn from truncated (0, Infinity) normal distributions, with different locations and scales for interspecific and intraspecific transmission (the latter always greater than the former) in each simulation run. For the competing species model, all *K* values and all self-competition values *α_i_*_=_ *_j_* were set to 1. Additionally, a host–host interaction network value *c* was drawn from a uniform distribution between 0 and 1. Then, each species pair with probability *c* had *α* values drawn from a uniform distribution between 0 and 1, otherwise they were set to 0. The number of mutualistic interactions in each simulation run is defined as the number of host species for which the growth rate of infected individuals is greater than uninfected individuals. After 1,000 generations, connectance as the proportion of realized host–plasmid links was calculated as the number of host populations with a symbiont prevalence of at least 0.005. This was an arbitrarily chosen cutoff for biologically significant links, as the presence of even one mutualist prohibits any species from reaching 0 symbiont prevalence due to constant interspecific transmission. Symbiont generality was calculated as *G* = 2*^H^*, with *H* being the Shannon diversity of symbiont prevalence across hosts

$$H=-\sum_{i=1}^{n}p_{i}\;\text{ln}\;p_{i},$$

where *n* is the number of links, and *p_i_* is the proportion of total community infection frequency attributable to species *i* (from the pool of species with a symbiont prevalence of at least 0.005).

### Bacterial Community.

The five bacterial species in our community were selected for in vitro coexistence from a range of soil isolates from a previous study (37). Based on 16S rDNA sequences, the isolates were most closely related to *Variovorax* sp., *Ochrobactrum* sp., *Pseudomonas* sp., *Stenotrophomonas* sp., and *Achromobacter* sp., respectively. Distinct colony morphologies of the five strains on King’s medium B (KB) agar allowed for rapid detection and enumeration of the five different species.

### Plasmid Introduction.

Plasmid donor strains were constructed for use in the diversity experiment. The self-transmissible, Incp-1ε–type plasmid pKJK5::gfp confers resistance to tetracycline and has a very broad host range (29). Plasmid-encoded resistance is mediated through the tetracycline efflux pump TetA without degradation and hence loss of activity of tetracycline. Therefore, no group protection is afforded to tetracycline-susceptible strains by resistant neighbors. Plasmid pKJK5 was introduced individually to each of the five species through conjugation in biparental matings with donor strain *Escherichia coli* MG1655::lacIq-pLpp-mCherry-KmR. Each strain was grown in 5 mL of unshaken lysogeny broth (LB) in a 1:1 ratio with the *E. coli* donor strain for 24 h at 28 °C and plated on solid minimal medium supplemented with 10 mM sodium citrate and 10 µg/mL tetracycline. Citrate as the sole carbon source counterselects against growth of the *E. coli* donor strain, while tetracycline selects for those recipient bacteria carrying the resistance plasmid. Successful conjugation was detected via expression of plasmid-encoded green fluorescent protein (GFP) via fluorescence stereomicroscopy. Individual green colonies were transferred to 5 mL of LB supplemented with 10 µg/mL tetracycline, grown for 24 h at 28 °C, and cryogenically frozen at −80° in 25% glycerol solution. All isolates and constructed strains used in this study can be found in *SI Appendix*, Table S1.

### Culture Conditions.

Bacterial communities were cultured under four growth conditions to determine the effect of the plasmid on bacterial diversity in the presence/absence of tetracycline. Communities were separated into four treatment groups: antibiotic (community plus tetracycline; *n* = 6), parasite (community plus plasmid in three different donor species; *n* = 18), mutualist (community plus plasmid supplied through one donor species and tetracycline; *n* = 17), and control (community without tetracycline or plasmid; *n* = 6). Communities were set up with equal densities of each of the five species in 25-mL glass microcosms containing 10 mL of 1/64th tryptic soy broth (TSB) incubated at 28 °C at 150 rpm. To the antibiotic and mutualist treatments, a final concentration of 0.2 µg/mL tetracycline was added. For plasmid and mutualist treatments, plasmid pKJK5 was introduced to the community by replacing one of the five original strains with its plasmid-hosting counterpart *Ochrobactrum* sp. (*n* = 6), *Pseudomonas* sp. (*n* = 6), or *Variovorax* sp. (*n* = 6). Every 7 d, 100 µL of culture was transferred into a fresh microcosm for a total of 5 wk. The experiment was limited to four transfers (∼50 generations) to minimize evolutionary change. After weeks 1, 3, and 5, the communities were plated onto KB agar for 48 h at 28 °C, and individual colonies were identified and counted.

### Identifying Transconjugants.

To build interaction networks for weeks 1 and 5, plasmid hosts were identified via green fluorescence. Colonies of both *Pseudomonas* sp. and *Ochrobactrum* sp. change visibly with the introduction of the plasmid. Host colonies were detected for these two species by excitation of agar plates with royal blue light at 440- to 460-nm wavelength viewed through a 500-nm filter from the NIGHTSEA stereo microscope fluorescence adapter kit (NIGHTSEA, LLC). Host colonies were not detectable using this method for the remaining three species. Instead, for each species within each replicate, 12 colonies (or as many as were available if less than 12) were picked and transferred to individual wells containing 50 µL of full-strength TSB supplemented with 0.2 µg/mL tetracycline in a 96-well plate. After 24 h of growth at 28 °C, green fluorescence was determined using a Synergy 2 microplate reader (Biotek; excitation filter = 485 to 520 nm and emission filter = 528 to 620 nm). Thus, the ratio of plasmid host colonies detected to all colonies tested was used to calculate edge weights in our network analysis.

### Network Analysis.

To understand how interaction type (mutualistic or parasitic) between plasmids and their hosts affect host–symbiont associations over time, we plotted networks based on mean numbers and mean infection rates for each plasmid treatment in weeks 1 and 5 of the experiment (*SI Appendix*, Fig. S4). Host abundance data were used from colony counts, while infection rate was estimated via green fluorescence. For plotting the networks, we used the “plotweb” function from the package “bipartite” (38) in R (39). To test how treatment impacts the generality of the plasmid, we estimated connectance (as the number of realized links divided by the number of all possible links) and the generality of the plasmid per replicate in week 5. For generality, we used *G* = 2*^H^*, with *H* being the Shannon diversity of interactions for the plasmid

$$H=-\sum_{i=1}^{n}p_{i}\;\text{ln}\;p_{i},$$

where *n* is the number of links, and *p_i_* is the total number of plasmid-hosting bacteria in the community divided by the number from species *i* (from the pool of species with at least a single detected plasmid). We then used connectance and plasmid generality as response variables to test for the impact of interaction type (presence of tetracycline) and plasmid donor species over time (weeks 1 and 5) by including the interaction between all three terms in the model. The network metrics were analyzed with linear mixed effects models based by including replicate ID as a random factor provided by the nlme package (40) and included an autocorrelation term to account for the nonindependence of repeated measures.

### Coevolution.

A microcosm from the mutualistic treatment of the main experiment with *Ochrobactrum* sp. as the donor and one from the parasitic treatment with *Pseudomonas* sp. as the donor were identified as the replicates that underwent the greatest change in connectance (positive and negative, respectively) between weeks 1 and 5. From each of these replicates and from the respective ancestral stock, six plasmid-hosting colonies of the donor species were isolated. The isolated colonies were then inoculated into communities and cultured for 1 wk under identical conditions as the parasitic treatment from the main experiment. The resulting microcosms were plated on both plain KB agar and selective KB + tetracycline agar plates. The presence of the plasmid in the donor species was identified by excitation of agar plates with royal blue light at 440- to 460-nm wavelength viewed through a 500-nm filter from the NIGHTSEA stereo microscope fluorescence adapter kit (NIGHTSEA, LLC), whereas transconjugants from other species were identified by comparing colony counts between selective and nonselective plates. A quasibinomial GLM was used to compare the rate of plasmid loss (i.e., the proportion of infected hosts after 1 wk) between ancestral and evolved *Pseudomonas* sp. Separate Poisson family GLMs were used to contrast the number of network links (species in which the plasmid was detected) between ancestral and evolved for each donor.

### Conjugation Assay.

To determine whether the concentration of tetracycline used in our experiment caused an increase in conjugation rates, we performed filter mating assays with and without tetracycline. This assay makes use of the GFP tag on pKJK5::GFP, which is suppressed in the plasmid donor *E. coli* MG1655. Potential plasmid recipients (the five species from the main experiment) are forced onto a two-dimensional surface surrounded by plasmid donors, ensuring that any green microcolonies that emerge are due to unique conjugation events. All ancestral strains were cultured for 48 h in 20 mL of full-strength TSB at 28 °C.

*E. coli* MG1655 + pKJK5 was grown under similar conditions with the addition of 1 µg/mL tetracycline to select against plasmid loss. All cultures were centrifuged for 30 min and resuspended in 50 mL of M9. Community strains were mixed and diluted 10-fold. The resulting community mixture was then mixed in equal volume with the *E. coli* suspension. Filter mating proceeded as described in ref. 41. Briefly, 2 mL of the mixed suspension was pumped through Cytiva Whatman Cyclopore polycarbonate black membrane filters. The filters were transferred to solid agar plates with 1/64th TSB as nutrient, six of which were supplemented with 0.2 µg/mL tetracycline. The inoculated plates were incubated at 28 °C for 3 h. Six images were taken of each filter at 10× magnification and 38.3-ms exposure with 400/20 excitation 508/20 emission filters.

Image manipulations and statistical analysis were performed in the Julia programming environment (35). Following background removal using Images.jl, microcolonies were counted via the fast scanning image segmentation algorithm implemented in ImageSegmentation.jl (42). The number of colonies detected per frame of view was compared using a hierarchical Poisson model as follows:

$$\begin{array}{c} y_{\mathrm{ijk}}∼\text{Poisson}\left(\theta_{ij}\right)\\ \theta_{ij}∼\text{Normal}(\alpha +\beta x_{i},\sigma_{i})\\ \alpha ∼\text{Normal}(500,500)\\ \beta ∼\text{Normal}(0,500)\\ \sigma_{i}∼\text{Exponential}(100), \end{array}$$

where *x* is an indicator variable denoting the presence of tetracycline, and *y_i,k_* is the green microcolony count in the *k*th image of the *j*th membrane filter in treatment *i*. The model was implemented in Turing.jl (43) using the No U-Turns (NUTS) algorithm with four chains of 1,000 iterations each (*R*^ˆ^ < 1.01 for all parameters).

### Growth Rates.

To determine the impact of the plasmid on each of the five species with and without antibiotics present, we estimated growth rate parameters for each species in monoculture. Approximately equal densities of each species were inoculated into 200 µL of 1/64th TSB with or without 0.2 µg/mL tetracycline in a 96-well plate. Cultures were grown for 48 h with optical density at 600 nm readings taken every 15 min. From the resulting data, we estimated *r* and *k* parameters of the logistic growth equation (44) $x(t)=\;\frac{k_{i}x_{0}}{x_{0}+(k_{i}-x_{0})e^{-r_{i}t_{j}}}$. We used only the intrinsic growth rate *r* as a measure of fitness, since *k* (and by extension *V*_max_) depends heavily on the response to optical density to cell density, which is affected by the plasmid. Our measurements are to be used as a proxy for individual fitness, whereas *k* relates more to the fitness of the population

$$\begin{array}{c} y_{ij}∼\text{Normal}\left(x_{ij},\sigma \right)\\ x_{ij}=\frac{k_{i}x_{0}}{x_{0}+(k_{i}-x_{0})e^{-r_{i}t_{j}}}\\ x_{0}∼\text{Half}\text{-}\text{laplace}(0,0.01)\\ r_{i}\;∼\text{Half}\text{-}\text{normal}(R,\sigma_{r})\\ k_{i}∼\text{Half}\text{-}\text{normal}(K,\sigma_{k})\\ \sigma ∼\text{Exponential}(1)\\ \sigma_{k}∼\text{Exponential}(1)\\ \sigma_{r}∼\text{Exponential}(1)\\ R∼\text{Half}\text{-}\text{normal}(0,1)\\ K∼\text{Half}\text{-}\text{normal}(0,1), \end{array}$$

where *y_ij_* is the *j*th optical density reading from the *i*th replicate, and *t_j_* is the *j*th time point. Separate models (each four chains of 2,000 iterations of the NUTS algorithm) were fit for each species/media/plasmid presence combination. To determine the effect of the plasmid for each species with and without tetracycline, we subtracted the *R* value from each posterior sample of growth with the plasmid from the corresponding sample without the plasmid. Overall, there is a clear benefit to plasmid carriage when tetracycline is present and cost when it is not. However, the costs are likely to be small compared to the benefits, and there is significant variation between the different species’ responses to the plasmid. In particular, *Variovorax* sp. derives little or no benefit from the plasmid, as it has good intrinsic tetracycline resistance. By contrast, *Ochrobactrum* sp., *Pseudomonas* sp., and *Stenotrophomonas* sp. receive a very large benefit due to their tetracycline susceptibility.

### Relative Abundance.

Relative abundance for each of the five bacterial species across all eight treatments was analyzed using generalized linear mixed models assuming a binomial error distribution and using the logit link function. As a response variable, we included a bivariate variable containing “abundance of species i” and “sum of abundance of other species” for each species. We further included treatment with eight levels as fixed factor and week and week squared as covariates. The quadratic term was added when improving the model fit and supported by small sample corrected Akaike information criterion (AICc) model selection (which was not the case for *Achromobacter* sp.). We further included an observation-level random factor to account for overdispersion in the data. The inclusion of replicate as random factor was not supported by AICc model selection. Temporal autocorrelation was negligible in this model (all partial autocorrelations below 0.2) and therefore were not included in the model. We used the function glmer from the R package lme4 (45) to fit this model.

Plasmid donor fitness was tested as relative abundance in the community over time. For each donor species, we ran a similar model as above but by including the factor donor with three levels (none, other, and focal) and the interaction with tetracycline (presence/absence) and week. As above, we included an observation-level random factor to account for overdispersion in the data. We also included a random slope for the week effect per replicate. The inclusion of replicate‐specific slopes was supported by AICc (delta = 9.54). To obtain 95% credible intervals for the model predictions, we used Bayesian methods provided by the function sim from the R package arm (46) to draw a random sample of 1,000 values from the joint posterior distribution of the model parameters. From these 1,000 sets of model parameters, predicted values were calculated, and their 2.5% and 97.5% quantiles were used as lower and upper limits of the 95% credible intervals.

### Total Abundance and Network Metrics.

We analyzed the impact of total cell density on both connectance and generality separately for week 1 and week 5, as it is not obvious that the effect (if any) should be the same in the short term as the long term. We used a hierarchical model structure to determine the effect of cell density within treatments (antibiotic presence X donor) to remove the confounds of donor identity and antibiotics. We used the following models for generality:

$$\begin{array}{l} G_{ij}∼\text{Normal}(y_{ij},\epsilon )\\ y_{ij}=\alpha_{i}+\beta_{i}\;x_{ij}\\ \alpha_{i}∼\text{Normal}(0,4)\\ \beta_{i}∼\text{Normal}(µ,\sigma )\\ µ∼\text{Normal}(0,1)\\ \epsilon ∼\text{Exponential}(1)\\ \sigma ∼\text{Exponential}(1), \end{array}$$

where $G_{ij}$ and $x_{ij}$ are the network generality and the colony count of the *j*th replicate of the *i*th treatment. We used the following model for connectance:

$$\begin{array}{c} C_{ij}∼\text{Binomial}(5,p_{ij})\\ p_{ij}=\text{logistic}(\alpha_{i}+\beta_{i}\;x_{ij})\\ \alpha_{i}∼\text{Normal}(0,4)\\ \beta_{i}∼\text{Normal}(µ,\sigma )\\ µ∼\text{Normal}(0,1)\\ \sigma ∼\text{Exponential}(1), \end{array}$$

where $C_{ij}$ and $x_{ij}$ are the connectance and the colony count of the *j*th replicate of the *i*th treatment. In each case, µ, the hyper-prior over the regression coefficients, is the parameter used to estimate the size and direction of the impact of cell density. In each case, we sampled the posterior using the NUTS algorithm, four chains of 5,000 iterations each.

## Supplementary Material

Supplementary File

## Data Availability

Data have been deposited in Zenodo at https://zenodo.org/record/5342448 and all code is available at GitHub (https://github.com/EvoArt/PlasmidNetworks). All other data are included in the manuscript and/or *SI Appendix*.
