# Supplementary material for: Fitness effects of plasmids shape the structure of bacteria–plasmid interaction networks
Source: Proc Natl Acad Sci U S A. 2022 May 25;119(22):e2118361119. doi: 10.1073/pnas.2118361119 (PMC9295774; doi:10.1073/pnas.2118361119)
Supplement: Supplementary File [file pnas.2118361119.sapp.pdf]

SUPPLEMENTARY MATERIALS

Summary of conjugation assay results

The mean estimate for the β parameter was -239.62 (with lower and upper 95 % quantiles = -331.41 and -151.71) displayed in Figure S2 on the scale of colonies /μm^2. Thus, in the presence of tetracycline a reduced the rate of conjugation events was detected. It is plausible that the reduced number of green fluorescent colonies is due to slowed metabolism, reducing the rate of GFP production. However, given these results, it is unlikely that the findings reported in the main text are due to an antibiotic-induced increase in conjugation rates.

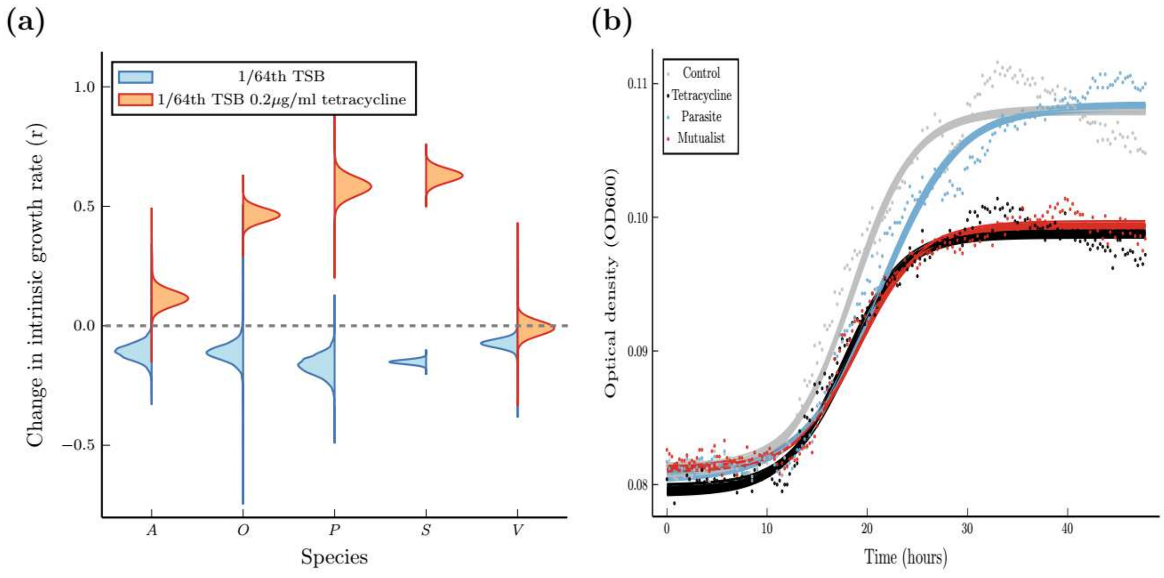

Figure S1. (A) Change in growth rate (r) affected by plasmid carriage for each of the 5 species, with or without tetracycline. Species identified by abbreviated genus name. **(B)** An example logistic growth equation fit to optical density data. Presented are results from 4

populations (1 for each treatment) of *Variovorax* sp.. Each line is calculated from growth rate parameters of one of 30 samples from the posterior distribution of the respective model.

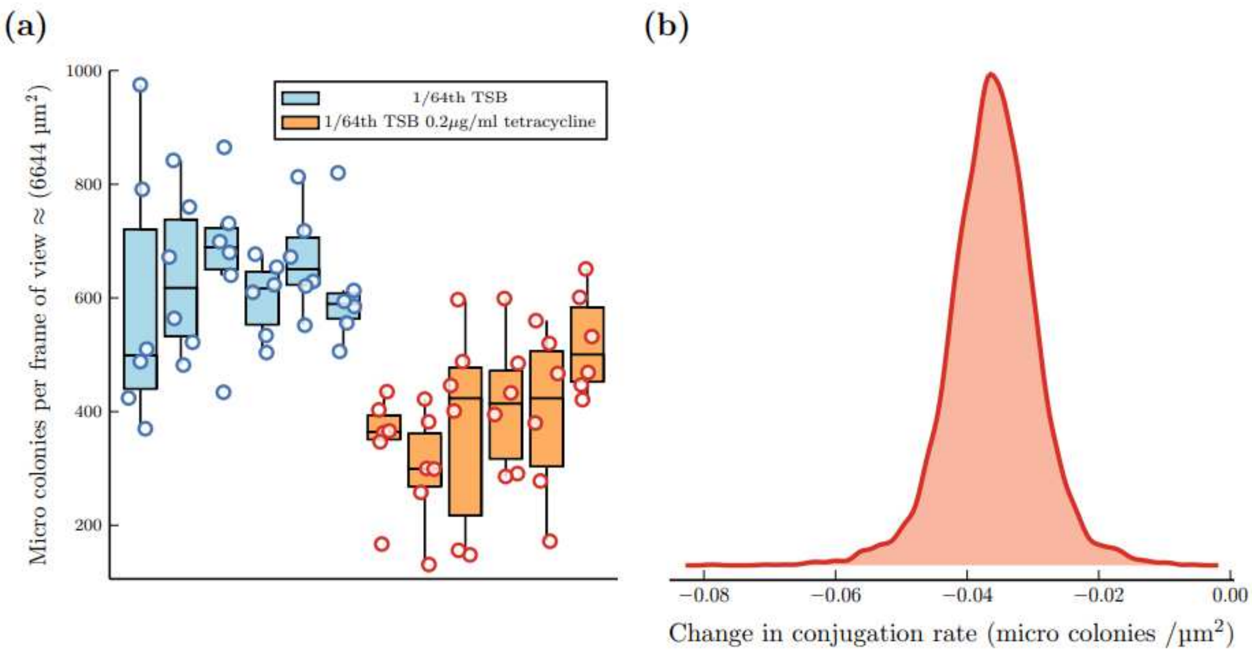

Figure S2. Results of filter mating conjugation assay. (A) Number of plasmid recipient micro-colonies detected via fluorescence microscopy on each replicate filter. Boxes indicate interquartile range and whiskers denote range of values across 6 images of the same filter. (B) Model results. Parameter estimates for change in rate of plasmid recipient micro-colony detection with the addition of tetracycline. Values have been transformed from original scale to colonies /μm^2.

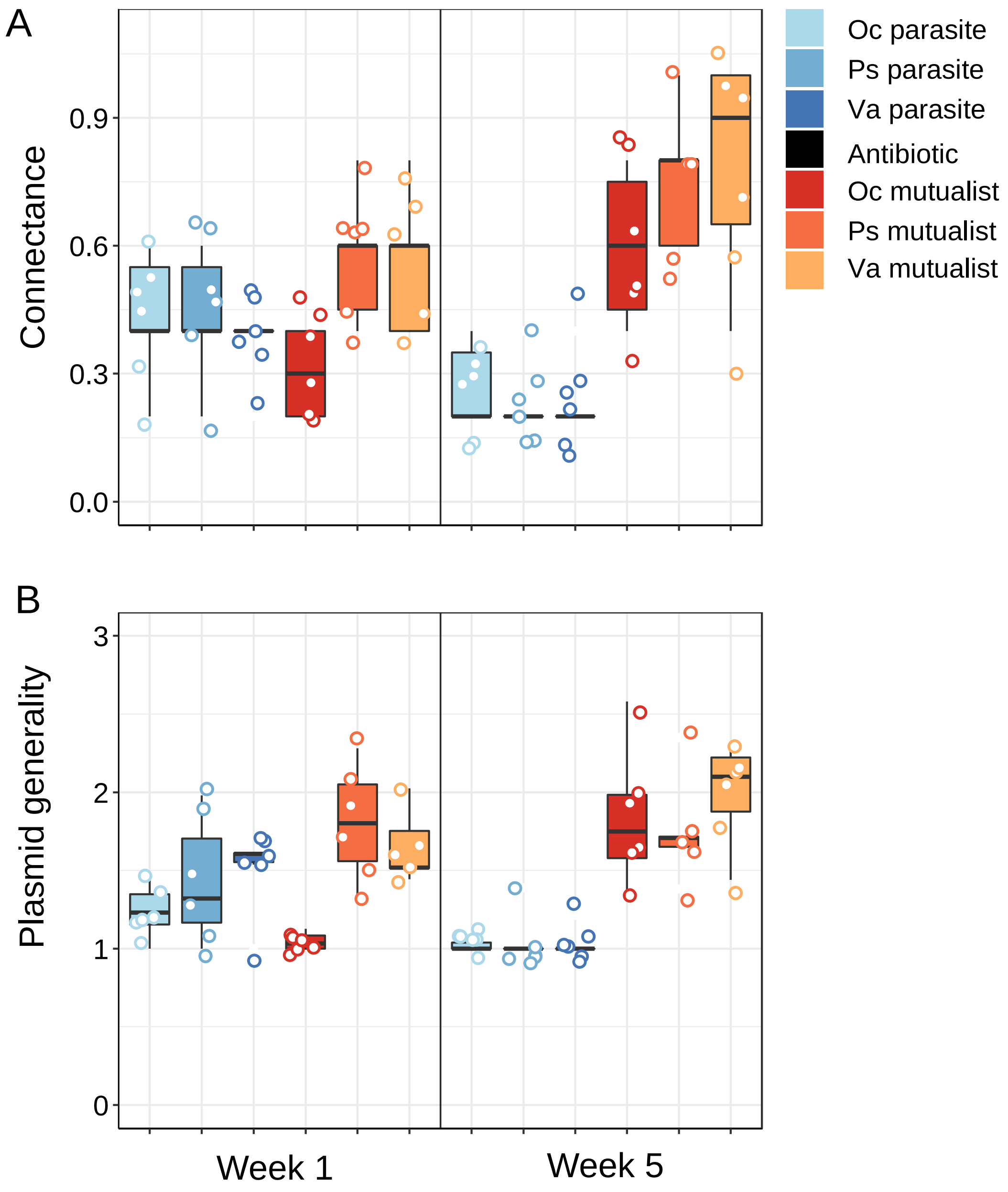

Figure S3. Network metrics for communities in week 1 and 5. are presented with median, interquartile ranges and single values for the different treatments (donor species identified by abbreviated genus name) with (b) connectance (realized number of links divided by the number of possible links) and (c) plasmid generality.

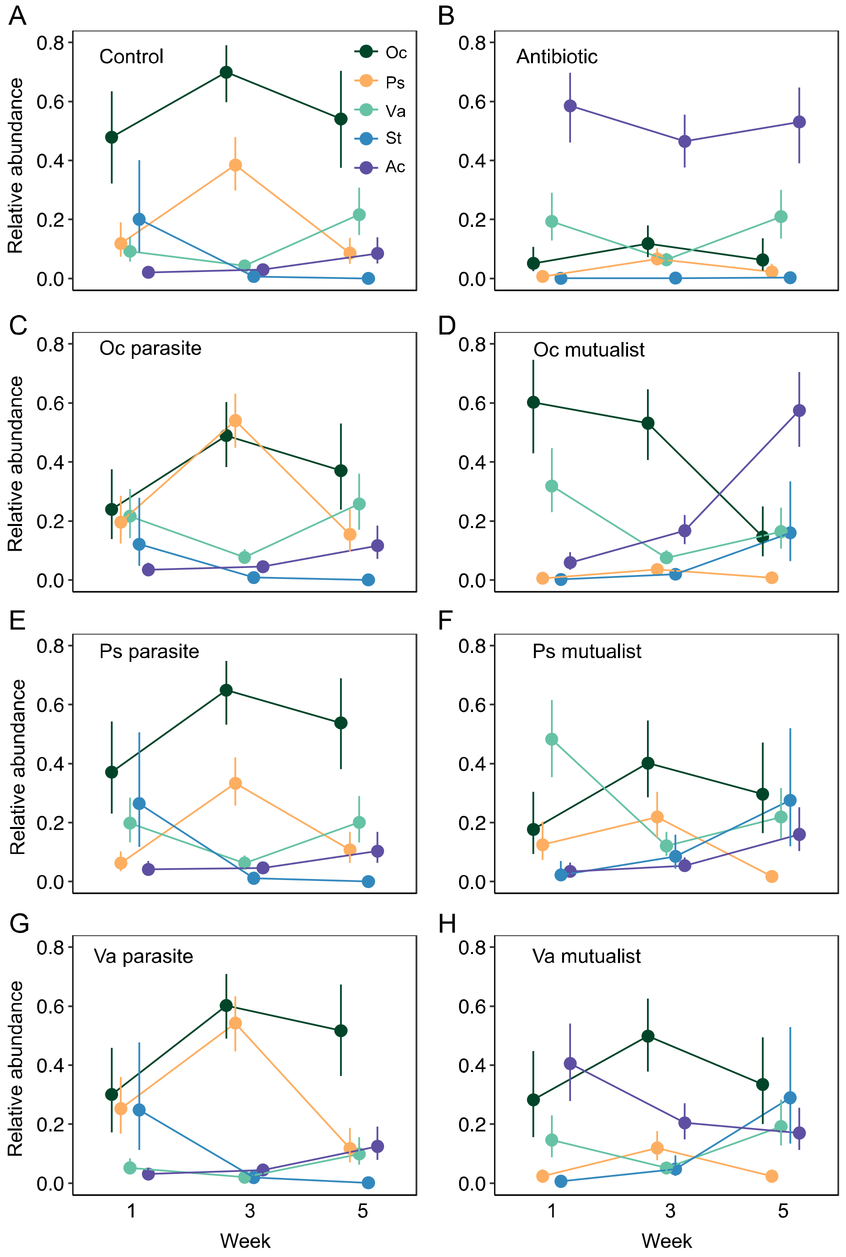

Figure S4. Relative abundance of bacterial species in the experimental communities. Relative abundances of all 5 species over time in control, antibiotic, mutualism, and parasitism treatments. Species identified by abbreviated genus name. *Ochrobactrum* sp., *Pseudomonas* sp. and *Variovorax* sp. served as plasmid donors in the respective treatments. Presented are mean and 95% credible intervals extracted from the posterior distribution of glmer models (see methods).

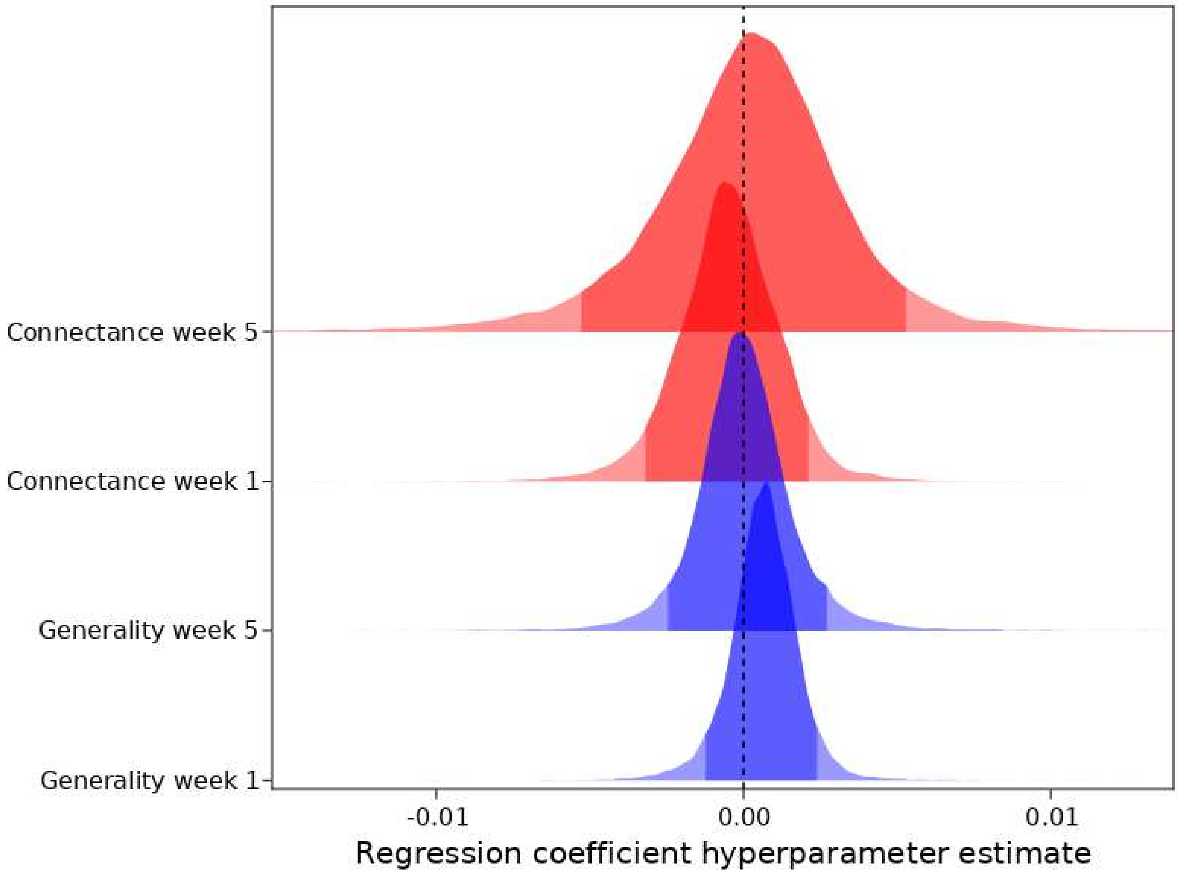

Figure S5. Effect of total community density on generality and connectance of bacteria-plasmid networks. Posterior estimates are shown for regression coefficient hyperparameter (μ) for each week/metric combination, with 95% credible intervals signified by darker shading. In each case, the credible interval spans 0. There is no clear evidence of either positive or negative effect of cell density on either network metric.

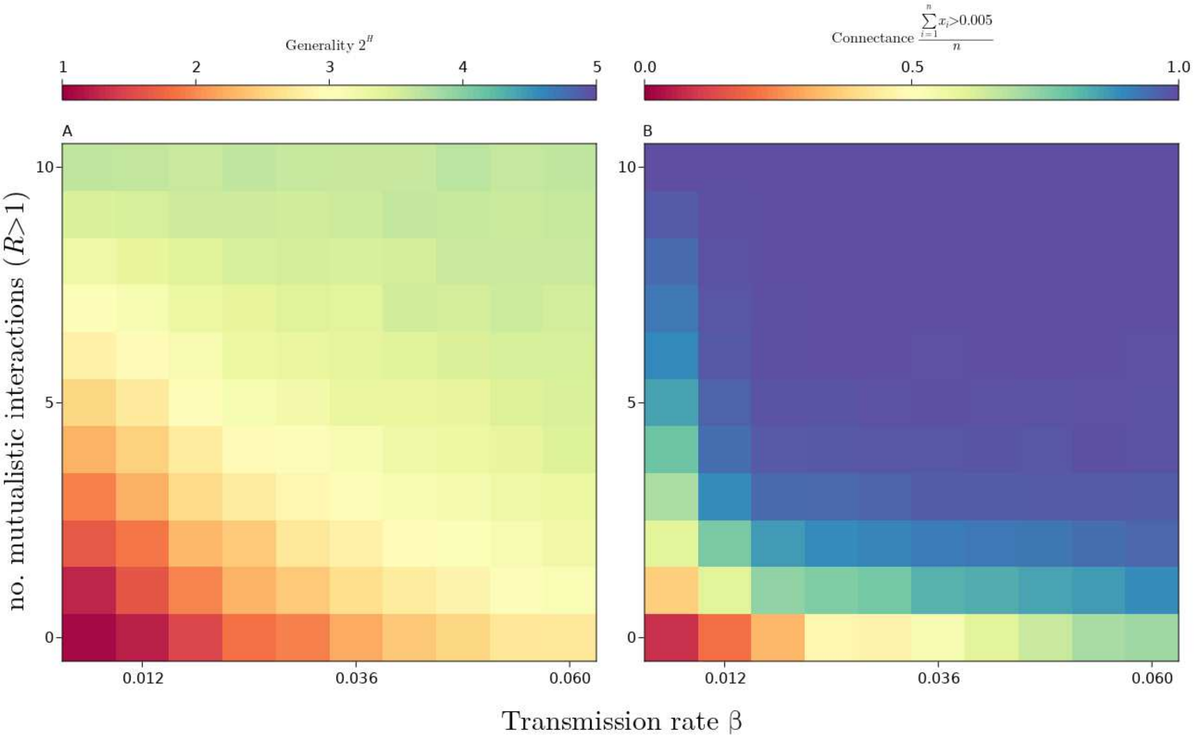

Figure S6. Results of numerical simulations where some host species dropped below an extinction threshold of $x_i < 0.005$. In these cases we do not count the extinct species' infection frequency towards the generality or (the numerator or denominator of the) connectance calculations. (A) Generality follows the same pattern as in Fig. 1, but with a general reduction due to the reduced number of hosts. (B) Connectance is also reduced as net horizontal transmission rates diminish with species extinctions.

Table S1. List of bacterial strains used in the study.

Strain	Plasmid	Reference
Variovorax sp.		(1)
Variovorax sp.	pKJK5::*gfp*, Tet^R	this study
Ochrobactrum sp.		(1)
Ochrobactrum sp.	pKJK5::*gfp*, Tet^R	this study
Pseudomonas sp.		(1)
Pseudomonas sp.	pKJK5::*gfp*, Tet^R	this study
Stenotrophomonas sp.		(1)
Stenotrophomonas sp.	pKJK5::*gfp*, Tet^R	this study
Achromobacter sp.		(1)
Achromobacter sp.	pKJK5::*gfp*, Tet^R	this study
E. coli MG1655::$lacI^q$-*pLpp-mCherry*-Km^R	pKJK5::*gfp*, Tet^R	(2)

References:

(1) Hesse E, O'Brien S, Tromas N, Bayer F, Luján AM, van Veen EM, Hodgson DJ, Buckling A. Ecological selection of siderophore-producing microbial taxa in response to heavy metal contamination. Ecol Lett. 2018 doi: 10.1111/ele.12878

(2) Klümper U, Riber L, Dechesne A, Sannazzarro A, Hansen LH, Sørensen SJ, Smets BF.

Broad host range plasmids can invade an unexpectedly diverse fraction of a soil bacterial community. The ISME journal 2015, doi:10.1038/ismej.2014.191
